# Fostering the Conversation About Complementary Medicine: Acceptability and Usefulness of Two Communication-Supporting Tools for Patients with Cancer

**DOI:** 10.3390/curroncol31110547

**Published:** 2024-11-20

**Authors:** Marit Mentink, Janneke Noordman, Anja Timmer-Bonte, Martine Busch, Sandra van Dulmen

**Affiliations:** 1 Department of Communication in Healthcare, Netherlands Institute for Health Services Research (Nivel), Otterstraat 118, 3512 CR Utrecht, The Netherlands; j.noordman@nivel.nl (J.N.); s.vandulmen@nivel.nl (S.v.D.); 2 Department of Primary and Community Care, Radboud Institute for Health Sciences, Radboud University Medical Center, Geert Grooteplein Zuid 21, 6525 EZ Nijmegen, The Netherlands; 3 Department of Medical Oncology, Radboud University Medical Center, Geert Grooteplein Zuid 10, 6525 GA Nijmegen, The Netherlands; anja.timmer-bonte@radboudumc.nl; 4 Van Praag Institute, Springweg 7, 3511 VH Utrecht, The Netherlands; mbusch@vanpraaginstituut.nl; 5 Faculty of Caring Science, University of Borås, Work Life and Social Welfare, Allégatan 1, 501 90 Borås, Sweden

**Keywords:** acceptability, cancer, patient–provider communication, complementary medicine, oncology, tools, usefulness

## Abstract

Both patients and providers experience barriers to discussing complementary medicine during oncology consultations. This study describes the development of two communication tools—a question prompt sheet and a visual slideshow—and aims to evaluate their acceptability, perceived usefulness, and intention to use among patients with cancer. Nine (former) patients with breast cancer were involved in the development of the tools as co-researchers. The 15-item evaluation questionnaire was completed by 144 participants recruited from three Dutch hospitals, a patient panel, and the Dutch Breast Cancer Society. The tools’ content and layout were generally acceptable, although suggestions were made to include items on exercise and diet in the question prompt sheet. About half of the participants found the tools useful, while the other half felt they were unnecessary, either because they could already discuss complementary medicine with their healthcare provider or had no interest in the topic. The tools were considered particularly helpful for fellow patients. The tools were well received though minor modifications were suggested. The lack of perceived need by half of the participants may have influenced the results. For effective use of the tools, it is important to identify patients who need extra support in discussing complementary medicine.

## 1. Introduction

Complementary medicine refers to non-mainstream approaches used together with conventional medicine, such as massage therapy, mindfulness, or dietary supplements [1]. The incorporation of complementary medicine alongside conventional cancer treatments has become increasingly prevalent among patients seeking to manage symptoms and side effects, enhance overall well-being, and exert a degree of control over their health [2]. Complementary medicine offers promising advantages for symptom relief, such as acupuncture to reduce chemotherapy-induced nausea and vomiting in breast cancer patients, or mindfulness-based interventions for patients with depression symptoms during or after cancer treatment [3,4,5]. Nonetheless, complementary medicine also presents inherent risks that demand careful consideration, such as interactions with conventional treatments [6,7]. For the delivery of effective and safe cancer care, patient–provider communication about complementary medicine is pivotal.

Patients with cancer are not always aware of the importance of disclosing complementary medicine use [8]. Additionally, they often feel hesitant to disclose complementary medicine use to their healthcare provider for reasons such as fear of disapproval or an expected lack of time or knowledge by the healthcare provider [9]. A systematic review showed that nondisclosure rates of complementary medicine ranged from 22% to 77% among patients with cancer [10]. Nonetheless, patients are the main initiators of discussions about complementary medicine during oncology consultations [11,12]. Important aspects of complementary medicine, such as its safety and the scientific evidence supporting its benefits, often go undiscussed [11]. Healthcare providers experience a lack of knowledge and confidence in adequately addressing complementary medicine during oncology consultations [13]. Fewer than 20% of oncology healthcare providers feel knowledgeable about complementary medicine [14]. When patients’ complementary medicine use is not discussed, healthcare providers may overlook patients’ needs. A previous study indicated that patients with cancer who use complementary medicine expressed more unmet needs compared to non-users, such as the need to be more involved in therapeutic choices or the need for better dialog with clinicians [15].

The barriers experienced by patients and healthcare providers lead to a gap in patient–provider communication about complementary medicine. Given the potential benefits and risks of complementary medicine, addressing this communication gap is crucial to encouraging safe and informed choices about complementary medicine use. Despite the fact that the impact of discussing complementary medicine on patient health outcomes has not been evaluated in previous studies, research has shown that oncology consultations that include discussions about complementary medicine are more patient-centered and result in higher satisfaction among both patients and healthcare providers [16].

Although several communication-supporting tools are available for patients with cancer in the Netherlands [17,18,19], to the best of our knowledge, there are no tools supporting patients in discussing complementary medicine. Therefore, two communication-supporting tools were developed for patients to guide them in introducing and discussing the topic of complementary medicine during oncology consultations. This study describes the development of these tools and aims to evaluate the acceptability, the perceived usefulness, and the intention to use these tools among patients with cancer.

## 2. Materials and Methods

A patient-participatory study design was used for the development and evaluation of two communication-supporting tools for patients with cancer. Nine co-researchers, consisting of (former) patients with breast cancer, collaborated with the research team throughout this study. The communication-supporting tools are the final products of a larger mixed-method research project focused on communication about complementary medicine in oncology titled ‘COMMON’ [20].

### 2.1. Development of the Communication-Supporting Tools

**Figure 1:**
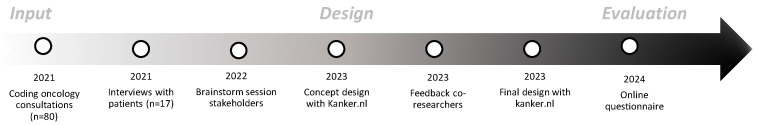
Steps in the development of communication-supporting tools.

The intervention mapping (IM) framework was used for the development of communication-supporting tools [21]. Prior to designing the tools, the needs of patients with cancer were assessed (Figure 1). First, audio-recorded consultations between patients with cancer and healthcare providers were analyzed [11]. If complementary medicine was discussed during the consultation, the section was coded with a custom observation scheme. Next, the experiences and needs of patients with communication about complementary medicine were assessed by conducting semi-structured interviews [22]. Subsequently, an online session was organized in which the results from earlier studies were presented and the attendees were invited to brainstorm about the tool contents. The session was attended by the research team, eight co-researchers, and members of several stakeholder parties: (1) the National Breast Cancer Society (BVN); (2) the Dutch Nursing Society (V&VN); (3) the Netherlands Comprehensive Cancer Organization (IKNL); and (4) an online information platform for Dutch patients with cancer (Kanker.nl).

A few key barriers to communication about complementary medicine emerged from the data that were collected, for instance, that not all patients with cancer are aware of the existence of complementary medicine or the importance of discussing complementary medicine use with their healthcare providers. In addition, not all patients with cancer are assertive enough to introduce the topic of complementary medicine to their healthcare provider. Furthermore, it is important for communication-supporting tools to be inclusive, e.g., by using visual information to support patients with lower literacy. The results clearly indicated the need among patients with cancer for support in conversations about complementary medicine. Therefore, the following two tools were developed:

1. A question prompt sheet (QPS) aims to stimulate and guide conversations, for example, in medical settings, by providing a list of prepared questions (i.e., question prompts) to individuals. The QPS we developed includes question prompts about complementary medicine for various situations that patients with cancer may encounter, such as having an interest in complementary medicine, already using it, or needing more information about it (Supplementary File S1). The question prompts were developed by the research team (M.M., S.v.D., J.N., A.T.B., and M.B.) and the medical editor of Kanker.nl, based on input gathered from observed consultations, interviews, and an online brainstorming session (Figure 1). The co-researchers were then asked for feedback on the draft question prompts, resulting in the addition of a few prompts and adjustments to sentence structures. The final QPS begins with an introductory text explaining what complementary medicine entails and the intended use of the QPS. This is followed by examples of symptoms and complementary therapies, after which twelve question prompts are provided. Examples of question prompts are: “I am being treated by an acupuncturist. Can I continue this during cancer treatment?” or “Does my hospital offer complementary medicine? If so, what is offered?”.
2. A slideshow that aims to highlight the importance of discussing complementary medicine with visual support (Supplementary File S1). The slideshow starts with giving a few examples of complementary medicine and for which symptoms they could be helpful. Then, it is highlighted that some types of complementary medicine can have side effects or interact with conventional treatment. Patients are recommended to discuss complementary medicine with their healthcare provider, and two examples are provided on how to initiate such a conversation. A reference is made to the QPS. The total number of slides is 14. The slides are supported by written and spoken text.

For the design of the content and layout of the communication-supporting tools, the research team collaborated with a medical editor and visual designer from Kanker.nl. The medical editor ensured that the tools were on language level B1 [23]. This website already provides information about the effects of complementary medicine for patients with cancer. Subsequently, the concept tools were presented to the co-researchers, who provided further feedback before the QPS and slideshow were finalized. It is our intention that the final version of the communication-supporting tools will become publicly available on the website of Kanker.nl.

### 2.2. Online Questionnaire

An online questionnaire was developed by researchers (S.v.D., J.N., and M.M.) to evaluate the tools. The initial version was piloted among four co-researchers, which led to the addition of response options and the rephrasing of a few questions. The final version consisted of 15 items (Supplementary File S2) that assessed the background characteristics of the participants (e.g., age, sex, education, type of cancer, and treatment phase) as well as how acceptable and useful they perceived the tools to be and their intention to use them. The majority of the questions were adapted from previous studies that evaluated comparable communication-supporting tools among patients with cancer [19,24].

#### 2.2.1. Acceptability

Acceptability refers to the extent to which users consider a healthcare intervention appropriate based on anticipated or experienced cognitive and emotional responses to the intervention [25]. In this study, acceptability is measured by:

- The first impression of the tool by selecting a maximum of three words best describing their feelings. Nine answer options were already provided (e.g., appealing, boring, confusing, and inviting). Participants could add other feelings in an open-answer category.
- The degree to which the tool was assessed as clear, helpful, comprehensive, professional, informative, reliable, simple, reassuring, or emotional on a 5-point scale.
- The attractiveness of the tool and appropriateness of the provided examples in the tool were scored on a 5-point Likert scale ranging from 1 (strongly disagree) to 5 (strongly agree).

#### 2.2.2. Perceived Usefulness

Perceived usefulness is a term extracted from the Technology Acceptance Model (TAM) [26] and can be defined as the degree to which a user sees the added value of a product. In this study, perceived usefulness is measured by:

- Eight statements about the perceived usefulness of the tool for patient–provider communication about complementary medicine; the educational value of the tool; and the perceived usefulness of the tools for other patients or relatives. The statements are scored on a 5-point Likert scale ranging from 1 (strongly disagree) to 5 (strongly agree).
- The compilation of the top three most useful questions (QPS only), including an open-ended category where participants could describe which questions were missing.

#### 2.2.3. Intention to Use

- The participants were asked about their intention to use the tool on a 5-point Likert scale ranging from 1 (strongly disagree) to 5 (strongly agree).
- Using a yes/no question, participants were asked whether they would use the QPS, including an open-ended category where participants could describe why they did not intend to use the QPS.

### 2.3. Participants and Recruitment

The eligibility criteria for participation in the evaluation questionnaire were (1) 18 years or older, (2) Dutch-speaking, and (3) currently or during the last 6 months in treatment for cancer. The aim was to include at least 90 participants. Oncology departments of three non-academic hospitals in the Netherlands were involved in the recruitment of participants. In each hospital, a study coordinator was appointed who ensured the distribution of a flyer with a call for study participation among patients visiting the clinic. The flyer included a link and QR code, which led to the online questionnaire. The call for study participation was also distributed online by the National Breast Cancer Society (BVN). In addition, patients with cancer who were members of a panel (*n* = 819) were e-mailed with a request for study participation. This panel is part of Kanker.nl.

### 2.4. Data Collection

Participants were able to participate in the evaluation study between mid-November 2023 and mid-January 2024. When opening the link to the questionnaire, participants were first presented with study information and were asked to sign an online informed consent form. Subsequently, background information was collected. To increase study validity, a video with a duration of 1:39 min explained the concept of complementary medicine to participants. Examples of complementary medicine used in the video were mindfulness, yoga, massage, music therapy, and acupuncture. The video was already publicly available on the website of Kanker.nl. Next, the QPS and slideshow were presented, with accompanying questions to evaluate each of the two tools.

### 2.5. Statistical Analysis

Stata version 16.1 was used to calculate descriptive statistics, such as mean, standard deviation, and percentages, considering background characteristics and evaluation measures. Open coding was used to analyze comments on open questions.

## 3. Results

In total, 144 participants completed the questionnaire. Background characteristics are presented in Table 1.

### 3.1. Evaluation of the Question Prompt Sheet (QPS)

#### 3.1.1. Acceptability

**Figure 2:**
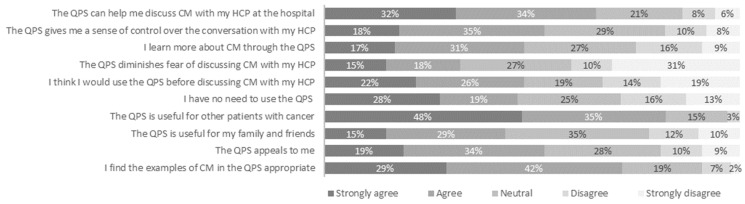
Acceptability and perceived usefulness of the question prompt sheet (QPS) for discussing complementary medicine (CM) with a healthcare provider (HCP) according to patients with cancer (*n* = 144).

At first impression, the QPS was described as clear (71%), professional (33%), or inviting (33%) by the participants. Upon further inspection, ≥70% of the participants rated the QPS as clear, easy, helpful, or professional. The QPS was described as not educational by 14% of the participants and as incomplete by 10% of the participants. As presented in Figure 2, approximately half of the participants (53%) found the QPS appealing and a majority of the participants (71%) perceived the examples in the QPS as appropriate.

#### 3.1.2. Perceived Usefulness

Figure 2 shows that the majority of the participants (66%) felt that the QPS could help them discuss complementary medicine with their healthcare provider by giving them a sense of control over the conversation (53%), learning more about the topic (48%), or diminishing fear of discussing complementary medicine (33%). Most participants (83%) found the QPS useful for fellow patients with cancer.

One-third of the participants (29%) found all question prompts useful. Two prompts were rated as most useful: (1) whether complementary medicine can be used for existing side effects (37%) and (2) whether complementary medicine can be used for potential symptoms (29%) (see Supplementary File S1). Participants rated the question prompt about where to find reliable complementary medicine practitioners as least useful (6%). In total, 7 out of 144 participants (5%) regarded none of the question prompts as useful. Sixteen participants (11%) missed question prompts in the QPS, such as question prompts about nutrition and exercise or about experiences of fellow patients with complementary medicine.

#### 3.1.3. Intention to Use

Approximately half of the participants (48%) indicated they would use the QPS (Figure 2). In total, 47% of participants felt they had no need to use the QPS. An additional yes/no question about the intention to use the QPS showed that 53% of participants would use the QPS and 47% of participants would not. Among those not intending to use the QPS, the most common reasons provided were as follows: (1) ability to discuss complementary medicine without support; (2) not interested in discussing complementary medicine with their healthcare provider (e.g., sufficiently informed themselves and lack of time or knowledge by healthcare provider); and (3) not interested in complementary medicine. Four participants mentioned reasons inherent to the layout or content of the QPS, such as “too crowded” or “missing examples of nutrition and exercise”.

### 3.2. Evaluation of the Slideshow

#### 3.2.1. Acceptability

At first impression, the participants described the slideshow as clear (67%), professional (30%), or inviting (26%). After taking a closer look, ≥70% of the participants perceived the slideshow to be clear, easy, reliable, and professional. In total, 43% of participants indicated finding the slideshow appealing, and 29% found the examples of complementary medicine used in the slideshow appropriate. Twenty-percent of the participants felt that the slideshow was incomplete, and 15% of participants indicated that the slideshow was slow and/or not educational. In an open question, patients indicated that they missed in-depth information about complementary medicine or that they felt the slideshow focused too much on symptoms or risks.

#### 3.2.2. Perceived Usefulness

**Figure 3:**
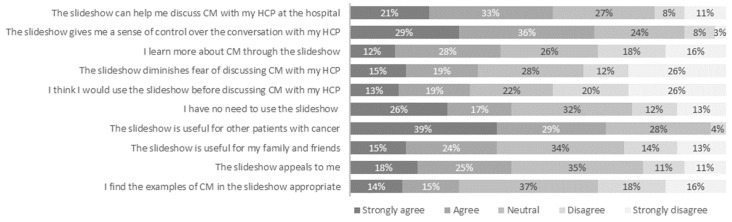
Acceptability and perceived usefulness of the slideshow for discussing complementary medicine (CM) with a healthcare provider (HCP) according to patients with cancer (*n* = 144).

Figure 3 shows that approximately half of the participants (54%) regarded the slideshow as helpful for discussing complementary medicine with their healthcare provider. In total, 65% of the participants indicated that the slideshow could provide them with a sense of control over the conversation with their healthcare provider. A minority of the participants felt that the slideshow taught them something about complementary medicine (40%) or diminished their fear of discussing the topic (34%). Most participants (68%) perceived the slideshow as useful for other patients.

#### 3.2.3. Intention to Use

Thirty-two percent of participants intended to use the slideshow (Figure 3). In total, 43% of participants felt that there was no need to use the slideshow.

### 3.3. Subanalyses Intention-to-Use Rates

The intention to use the tools among male participants seems to be higher compared to female participants (see Table 2). In addition, participants with a lower education level seem to be intended to use the tools more often compared to higher-educated participants. Current CM users seem less inclined to use the tools compared to current non-users.

### 3.4. Tool Revisions

The results of the evaluation questionnaire were discussed within the research team and presented to the medical editor and visual designer of Kanker.nl. The main revision made based on the evaluation results was the clarification of the purpose of using the QPS: “Use this conversation aid before and during a discussion with your doctor or nurse. This will help you prepare and organize your questions. The questions are examples. You can always modify them or add your own”. Most of the additional information suggested by participants was already available within the tools or on the Kanker.nl platform.

## 4. Discussion

The evaluation of the tools indicated that participants were neutral to positive in their acceptance of the QPS and the slideshow. The tools were identified as clear, easy, and reliable by most participants. Some areas of improvement in the tool content emerged, such as the desire to incorporate question prompts about diet and exercise in the QPS. Approximately half of the participants perceived the tools as useful for themselves, although the other half of the participants had no intention of using the QPS or slideshow. Several patients felt that they could adequately discuss the topic of complementary medicine without support. The tools were considered especially useful for fellow patients.

The intention-to-use rates among participants in this study (32% and 53%) were comparable to those reported in a review on the use of question prompt lists in health consultations in general [28] but slightly lower than the 75% intention-to-use rates of communication tools in oncology settings [19,29]. The main reason participants did not intend to use the QPS was their ability to discuss the topic of complementary medicine without the support of a tool. This trend has also been observed in previous evaluations of communication tools among patients with cancer, where patients may not feel the need for such aids personally but recommend them for other patients [24,30]. The overrepresentation of higher educated participants in the current study might account for this decreased need for the use of these tools. Indeed, our subanalyses showed decreased intention-to-use rates in highly educated participants. In a previous study, it was shown that patients with higher education are more inclined to disclose complementary medicine use to their oncologist [31]. Patients with a higher education level may be more assertive in patient–provider communication than those with a lower education level are. Research has shown that [32], patients can evolve from three communication states during oncology consultations: (1) overwhelmed and passive; (2) proactive and self-motivated; and (3) proficient and empowered. Communication-supporting tools are especially useful for helping patients transition from the first to the second state, i.e., from contributing little to the conversation to preparing their consultations to fulfill certain goals. The patients in the current sample were probably already in a proactive or proficient state of communication, given that the majority were members of a patient panel or society. Male participants seemed more intended to use the communication-supporting tools compared to female participants, which is in line with results of previous research in which male patients had lower disclosure rates of complementary medicine [31]. Surprisingly, patients who are currently not using complementary medicine are mostly inclined to use communication-supporting tools. A possible explanation could be that non-users may view communication-support tools as valuable resources to clarify their preferences or understand potential topics of discussion such as complementary medicine during consultations. Conversely, patients who already use complementary care might feel less need for these tools, having established alternative sources of support or information outside the clinical setting. Future research should further examine which patient characteristics are associated with the need for extra support in discussing complementary medicine to ensure targeted dissemination of the communication-supporting tools.

Some participants did not wish to discuss the topic of complementary medicine use with their healthcare provider because they felt sufficiently informed about the topic or perceived their healthcare provider to lack knowledge or time to discuss the topic. These results indicate that awareness about the importance of patients discussing complementary medicine use with healthcare providers should increase to avoid complementary medicine harming patients during conventional treatment [6,7]. In fact, the developed slideshow aimed to portray the importance of discussing complementary medicine in a visual way (it is important to note that participants were not presented with the slideshow until after they had provided a reason for their lack of intention to use the QPS). Other participants indicated that they were not interested in complementary medicine use itself and therefore had no intention of using the QPS. In the study sample, non-users of complementary medicine were overrepresented, although many of these non-users were former users or were interested in complementary medicine. Nonetheless, the overrepresentation of non-users could have contributed to the relatively high number of neutral responses to the questions involving a Likert scale, given that non-users have less of an opinion about the tools.

To evaluate the effectiveness of the communication-supporting tools in a clinical setting, this study could be succeeded by a randomized trial. For instance, characteristics of the consultation such as disclosure of complementary medicine, consultation satisfaction, or patient health outcomes could be compared between those who used a communication-supporting tool to prepare a conversation about complementary medicine and those who did not.

### 4.1. Study Limitations

The developed communication-supporting tools were intended for use in the generic population of patients with cancer. However, the results showed that several of the included participants in the current study did not feel the need to use the tools. It would have been appropriate to assess which patient characteristics are associated with the need for support in communication about complementary medicine, such as sex, type of cancer, or education level, prior to recruitment of participants for evaluation. 

The currently used method of convenience sampling can lead to selection bias. For instance, patients with lower education levels appeared to be underrepresented. It is conceivable that predominantly assertive or experienced patients acted on the call for study participation distributed by the patient panel and the Dutch Breast Cancer Society. Furthermore, the hospitals from which participants were recruited already launched initiatives in the field of integrating complementary medicine into oncological care, which could have led to the recruitment of participants who were better informed about complementary medicine or who were experiencing fewer barriers to discussing complementary medicine with their healthcare provider. Additionally, collecting data online inadvertently excluded patients who were not digitally proficient.

### 4.2. Clinical Implications

In clinical oncology, choice of treatment and patient information are individually tailored. Patients selecting personally relevant questions from a QPS can enhance tailored communication during oncology. This tool can empower patients by giving them a structured way to initiate conversations about complementary medicine. The results of this study showed that the QPS and slideshow seemed particularly valuable to patients who are unaware of the importance of discussing complementary medicine with their healthcare provider or to patients who experience barriers to talking about the topic with their healthcare provider. The slideshow can raise awareness of the importance of discussing complementary medicine among less-informed patients. The QPS ensures that patients who are in need of guidance receive necessary support to engage in meaningful discussions about complementary medicine. For patients with cancer uninterested in complementary medicine, the tools can at best raise awareness about the existence and potential benefits and risks of complementary medicine and the importance of discussing complementary medicine use with their healthcare provider.

The responsibility for discussing the topic of complementary medicine use during oncology consultations should not lie solely with patients. Given that communication is reciprocal, healthcare providers also play an important role. The extent to which healthcare providers prioritize patient-centered communication and patient involvement—such as by using communication tools—can potentially influence outcomes such as patients’ trust in their healthcare providers and their evaluation of healthcare quality [33]. Healthcare providers should be aware that some patients perceive them as lacking knowledge and time to adequately discuss complementary medicine. In addition, a reason for nondisclosure of complementary medicine among patients is a lack of inquiry by the healthcare provider [8]. Therefore, it is important that healthcare providers adopt an active role in initiating the subject of complementary medicine, especially towards patients who are known to have decreased complementary medicine use disclosure rates, such as patients with lower education and male patients [31]. However, many healthcare providers feel underprepared to have these conversations, with fewer than 20% feeling confident in their knowledge of complementary medicine [14]. To reinforce knowledge and confidence among healthcare providers, in future research we aim to develop tools that support healthcare providers in confidently initiating and managing discussions about complementary medicine.

## 5. Conclusions

The question prompt sheet and slideshow were generally accepted by the participants, although the participants demanded minor alterations or additions to the content of the tools. Approximately half of the participants felt no need for the use of the tools, which probably influenced the results of this evaluation study. To optimize the use of the tools, it is important to assess which patient characteristics are associated with the need for support in discussing complementary medicine.

## Figures and Tables

**Table 1:** Background characteristics of participants (*n* = 144).

Variable	M (SD), Range	
Age in Years	58 (12.2), 27–83	
	*N*	(%)
Sex		
Male	54	(36%)
Female	90	(63%)
Education level according to ISCED 2011 [27]		
Low	13	(9%)
Medium	47	(33%)
High	84	(58%)
Type of cancer (multiple answers possible)		
Breast	62	(43%)
Urological	30	(21%)
Gastrointestinal	18	(13%)
Skin	14	(10%)
Blood or lymph	12	(8%)
Gynecological	8	(6%)
Lung	8	(6%)
Head or neck	6	(4%)
Other	4	(3%)
Treatment status		
In active treatment	82	(57%)
Post-treatment (≥6 months)	41	(28%)
Other ^a^	21	(15%)
Current complementary medicine (CM) user		
Yes, CM use discussed with HCP ^b^	34	(24%)
Yes, CM use not discussed with HCP	19	(13%)
No, but interested in CM or former user	61	(42%)
No, not interested in CM	30	(21%)
Recruited through		
Patient panel	92	(64%)
Hospital	27	(19%)
Breast cancer society	25	(17%)

The percentages may add to less or more than 100% due to rounding. ^a^ E.g., awaiting treatment, regular check-ups, wait-and-see, and palliative treatment. ^b^ Healthcare provider.

**Table 2:** Intention-to-use rates according to sex, education level, and complementary medicine use (*n* = 144).

Variable	*n*	Intention to Use QPS (%)	Intention to Use Slideshow (%)	Mean Intention-to-Use Rate (%)
Sex				
Male	54	59%	39%	49%
Female	90	49%	27%	38%
Education level				
Low	13	76%	38%	57%
Medium	47	60%	38%	49%
High	84	45%	27%	36%
Current complementary (CM) user				
Yes, CM use discussed with HCP	34	47%	24%	36%
Yes, CM use not discussed with HCP	19	47%	21%	34%
No, but interested in CM or former user	61	64%	36%	50%
No, not interested in CM	30	40%	40%	40%
Total population	144	53%	32%	43%

## Data Availability

The data that support the findings of this study are available from the corresponding author upon reasonable request.

## Supplementary Materials

The following supporting information can be downloaded at: https://www.mdpi.com/article/10.3390/curroncol31110547/s1. Supplementary File S1: Impression of tools (in Dutch). Supplementary File S2: Evaluation questionnaire (translated to English).
