# Supplementary material for: Fostering the Conversation About Complementary Medicine: Acceptability and Usefulness of Two Communication-Supporting Tools for Patients with Cancer
Source: Curr Oncol. 2024 Nov 20;31(11):7414–25. doi: 10.3390/curroncol31110547 (PMC11592416; doi:10.3390/curroncol31110547)
Supplement: Supplementary file 1 [file curroncol-31-00547-s001.zip › Supplementary File S1-impression of tools in Dutch.pdf]

Supplemental File S1 – Communication-supporting tools

Question Prompt Sheet (QPS)

Gespreksshulp

In gesprek met je arts of verpleegkundige over complementaire zorg (aanvullende zorg)

Complementaire zorg kan je misschien helpen om je lichamelijk of geestelijk beter te voelen, tijdens of na je behandeling. Deze zorg kun je aanvullend op de reguliere zorg krijgen. Goed om te weten: complementaire zorg kan kanker niet genezen.

Gebruik deze gesprekshulp voor en tijdens een gesprek met je arts of verpleegkundige. Zo kun je je goed voorbereiden en heb je je vragen op een rijtje. De vragen zijn voorbeelden. Je kunt ze altijd aanpassen of eigen vragen toevoegen.

Hier heb ik last van

- ☐ Angst en spanning
- ☐ Somberheid, depressie en stemmingswisselingen, vermoeidheid
- ☐ Misselijkheid en overgeven
- ☐ Pijn
- ☐ Vermoeidheid, slaapproblemen
- ☐ Opvliegers
- ☐ Anders, namelijk …..

Voorbeelden van complementaire zorg

- ☐ Acupunctuur
- ☐ Aromazorg
- ☐ Beeldende therapie
- ☐ Haptotherapie
- ☐ Homeopathie
- ☐ Hypnotherapie
- ☐ Massage
- ☐ Medicinale cannabis
- ☐ Muziektherapie
- ☐ Ontspanningsoefeningen
- ☐ Therapeutic Touch
- ☐ Vaktherapie
- ☐ Voedingssupplementen
- ☐ Yoga
- ☐ Anders, namelijk ……

kanker.nl

Gesprekshulp complementaire zorg

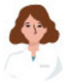

Vragen die je kunt stellen aan je arts of verpleegkundige

Als je benieuwd bent of complementaire zorg je kan ondersteunen bij klachten

Ik heb last van ....
[vul in welke klachten je hebt, bijvoorbeeld vermoeidheid, pijn, opvliegers]

Is daar iets aan te doen, misschien met complementaire zorg?

Stel dat ik bijwerkingen krijg van de behandeling. Is daar iets tegen te doen zonder medicijnen? Bijvoorbeeld met een vorm van complementaire zorg?

Als je al complementaire zorg gebruikt

Ik gebruik ....
[vul soort complementaire zorg in, bijvoorbeeld voedingssupplementen, cannabis of essentiële oliën]

Kan ik hiermee doorgaan tijdens de behandeling tegen kanker?

Ik doe aan ....
[vul soort complementaire zorg in, bijvoorbeeld yoga, Tai Chi].

Kan ik hiermee doorgaan tijdens de behandeling tegen kanker?

Ik ben onder behandeling van een ....
[vul de soort complementaire behandelaar in, bijvoorbeeld een acupuncturist, een natuurgeneeskundige, een haptotherapeut]

Kan ik hiermee doorgaan tijdens de behandeling tegen kanker?

Notities

Gesprekshulp complementaire zorg

Vragen die je kunt stellen aan je arts of verpleegkundige

Als je erover nadenkt om complementaire zorg te gaan gebruiken

Ik wil misschien .... gebruiken.
[vul soort complementaire zorg in, bijvoorbeeld acupunctuur of kruidenproducten]

Kan dat kwaad?

Ik wil misschien .... gaan doen.
[vul soort complementaire zorg in, bijvoorbeeld mindfulness of massagetherapie]

Kan dat kwaad?

Als je meer informatie en ondersteuning wil op het gebied van complementaire zorg

Bij wie in dit ziekenhuis kan ik terecht voor meer informatie en/of ondersteuning over complementaire zorg?

Biedt mijn ziekenhuis zelf ook complementaire zorg aan? Zo ja, wat dan?

Vergoedt mijn zorgverzekeraar .... ?
[vul de soort complementaire zorg in]

Als ik zelf informatie wil zoeken over ...., wat zijn dan betrouwbare bronnen?
[vul de soort complementaire zorg in]

Waar vind ik een betrouwbare ....?
[vul de soort complementaire zorgverlener in die je zoekt, bijvoorbeeld haptotherapeut]

Staat jouw vraag niet tussen? Schrijf dan hier je vraag.

Tip Bedenk voor jezelf welke 2 of 3 vragen je in ieder geval wilt stellen.

Meer informatie vind je op kanker.nl/complementairezorg

kanker.nl

Slideshow

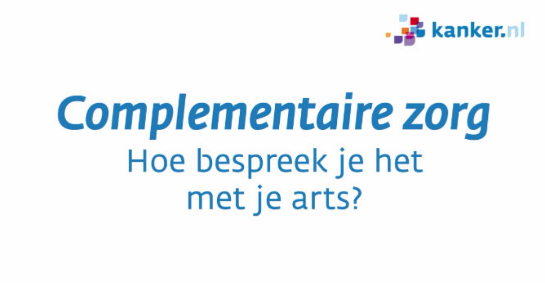
kanker.nl
Complementaire zorg
Hoe bespreek je het met je arts?

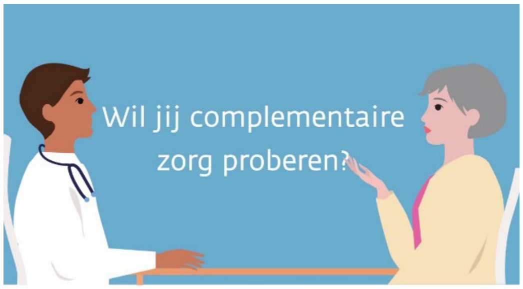
Wil jij complementaire zorg proberen?

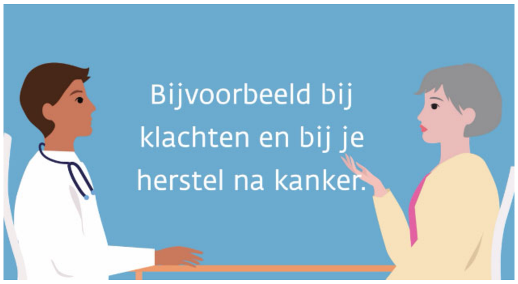
Bijvoorbeeld bij klachten en bij je herstel na kanker.

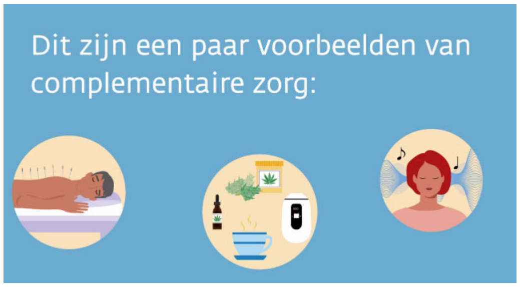
Dit zijn een paar voorbeelden van complementaire zorg:

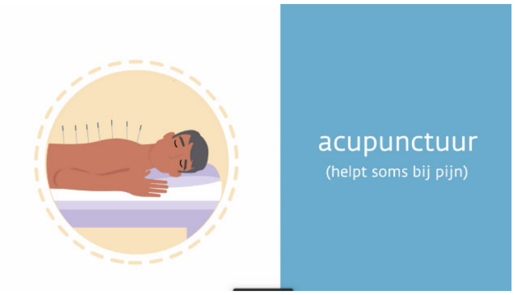
acupunctuur
(helpt soms bij pijn)

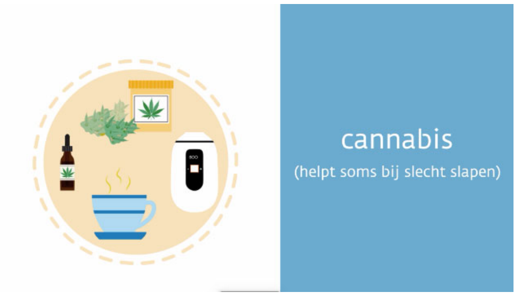
cannabis
(helpt soms bij slecht slapen)

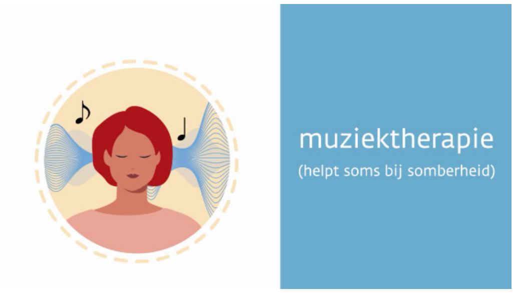
muziektherapie
(helpt soms bij somberheid)

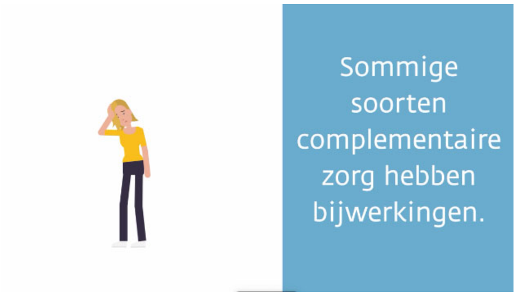
Sommige soorten complementaire zorg hebben bijwerkingen.

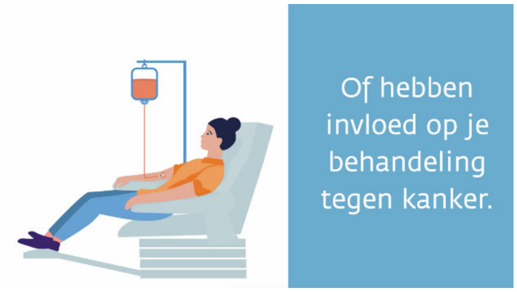
Of hebben invloed op je behandeling tegen kanker.

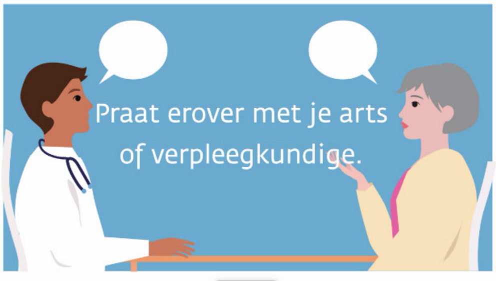
Praat erover met je arts of verpleegkundige.

Zeg bijvoorbeeld:

Ik slaap slecht.
Mag ik cannabis
gebruiken?

Of zeg:

Ik wil acupunctuur
proberen tegen pijn. Is dat
een goed idee?

In de gesprekshulp complementaire zorg vind je meer voorbeeldvragen.

kanker.nl/complementaire-zorg

We zijn er voor je. kanker.nl
