# Supplementary material for: Fostering the Conversation About Complementary Medicine: Acceptability and Usefulness of Two Communication-Supporting Tools for Patients with Cancer
Source: Curr Oncol. 2024 Nov 20;31(11):7414–25. doi: 10.3390/curroncol31110547 (PMC11592416; doi:10.3390/curroncol31110547)
Supplement: Supplementary file 1 [file curroncol-31-00547-s001.zip › Supplementary File S2 - evaluation questionnaire translated to English.pdf]

Supplemental File S2 – English translation of questionnaire pilot communication tools oncology

Demographics

1. What is your age (in years)?

__

2. What is your sex?
o Male
o Female
o Other

3. What is the highest education you completed?
o Primary/elementary school
o Primary or preparatory vocational education (such as LTS, LEAO, LHNO, VMBO)
o Pre-vocational secondary education (such as MAVO, (M)ULO, VMBO-t)
o Secondary vocational education. (such as MTS, MEAO)
o Senior general secondary or pre-university education (such as HAVO, VWO, HBS, MMS)
o Higher professional education (such as HBO, HTS, HEAO, HBO-V)
o University education
o Other, namely..

4. What type of cancer do you have or have had (multiple answers are possible)?
o Breast cancer (mammary carcinoma)
o Lung cancer
o Stomach, liver or colon cancer, including esophageal cancer, rectal cancer, pancreatic cancer (gastroenterological tumor)
o Gynecological cancer (e.g., cervical cancer, ovarian cancer)
o Blood or lymph node cancer (e.g., (non)-Hodgkin's disease, leukemia)
o Urological cancer (e.g., kidney, prostate, bladder cancer)
o Skin cancer (e.g., melanoma)
o Other type of cancer, namely...

5. Which situation applies to you?
o I am awaiting treatment (period between diagnosis and treatment)
o I am currently being treated for cancer
o I have completed cancer treatment in its entirety
o Other, namely.........

Complementary medicine

Complementary medicine literally means supplemental care. It adds something to regular anti-cancer treatment, such as chemotherapy or radiation. Complementary medicine can help with symptoms and recovery. Some examples of complementary medicine include: acupuncture, mindfulness, vitamin supplements, yoga. Complementary medicine is not the same as alternative medicine that replaces regular treatment.

Below is a video explaining what complementary medicine is.

VIDEO

6. Are you currently using complementary medicine?
- Yes, I am currently using complementary medicine
  - o If yes, do you discuss this use of complementary medicine with your doctor or nurse at the hospital?
    - ▪ Yes
    - ▪ No, because....

- No, but I have used complementary medicine in the past (for instance before you were diagnosed with cancer)
- No, but I have interest or need
- No, I have no interest or need

Below is a link to the conversation aid. This is a tool that patients can use to discuss complementary medicine with their health care provider. Click on the link to view the conversation aid. Then you can answer the questions about the conversation aid.

QPS

7. What is your first impression of the conversation aid?
Choose the word that best represents your feelings, or type in your own word. Maximum of 3 answers possible.

o Clear
o Professional
o Crowded
o Attractive
o Boring
o Confusing
o Reliable
o Gloomy
o Inviting
o Fill in a word of your own:..................................

8. Please indicate the extent to which you agree or disagree with the statements below. We would like to have your opinion. There are no right or wrong answers.

	**Totally disagree**				**Totally agree**
**1. The QPS can help me discuss complementary medicine with my healthcare provider at the hospital**	-	-	-	-	-
**2. I learn more about complementary medicine through the QPS**	-	-	-	-	-
**3. I have no need to use the QPS**	-	-	-	-	-
**4. I find the examples of complementary medicine in the QPS appropriate**	-	-	-	-	-
**5. The QPS gives me a sense of control over the conversation with my healthcare provider**	-	-	-	-	-
**6. I think I would use the QPS before discussing complementary medicine with my healthcare provider**	-	-	-	-	-

**7. The QPS diminishes fear of discussing complementary medicine with my healthcare provider**	-	-	-	-	-
**8. The QPS appeals to me**	-	-	-	-	-
**9. The QPS is useful for my family and friends**	-	-	-	-	-
**10. The QPS is useful for other patients with cancer**	-	-	-	-	-

9. The conversation aid is...
On each line, click the circle closest to your opinion.

Clear	o	o	o	o	o	Unclear
Useful	o	o	o	o	o	Not useful
Complete	o	o	o	o	o	Incomplete
Amateuristic	o	o	o	o	o	Professional
Educational	o	o	o	o	o	Not educational
Reliable	o	o	o	o	o	Unreliable
Difficult	o	o	o	o	o	Simple
Reassuring	o	o	o	o	o	Distressing
Emotional	o	o	o	o	o	Not emotional

10.Would you use the conversation aid to discuss complementary medicine with your health care provider?

- Yes

- No, because….

11. Which questions from the conversation aid, repeated below, do you find most useful? Choose up to 3.

- I do not find any of the questions useful.
- I suffer from ..... [fill in what symptoms you have, e.g., fatigue, pain, hot flashes]. Is there anything we can do about this, perhaps with complementary medicine?
- Suppose I get side effects from treatment. Is there anything that can be done without medication? For example, with some type of complementary medicine?
- I use .... [fill in type of complementary medicine, e.g. dietary supplements, cannabis or essential oils]. Can I continue this during cancer treatment?
- I do .... [insert type of complementary care, e.g. yoga, Tai Chi]. Can I continue this during cancer treatment?
- I am under the treatment of a .... [insert type of complementary practitioner, e.g. acupuncturist, naturopath, haptotherapist]. Can I continue this during cancer treatment?
- I may want to use .... [insert type of complementary medicine, for example, acupuncture or herbal products]. Can that hurt?
- I may want to do .... [fill in type of complementary medicine, for example, mindfulness or massage therapy]. Can that be harmful?
- Who in this hospital can I go to for more information and/or support about complementary medicine?
- Does this hospital itself offer complementary medicine? If so, what is offered?
- Does my health insurance company reimburse ....? [insert type of complementary medicine]
- If I want to search information about .... myself, what are reliable sources? [insert type of complementary medicine]

- Where can I find a reliable ....? [fill in the type of complementary medicine practitioner you are looking for, e.g. haptotherapist]
- I find all the questions useful

12. Are you missing any questions in the conversation aid that you would like to ask your health care provider about complementary medicine?

- Yes, namely.....
- No
-

Below is a slideshow about discussing complementary medicine with health care providers in the hospital. After watching the slideshow, please answer the questions.

SLIDESHOW

13. What is your first impression of the slideshow?
Choose the word that best represents your feeling, or type in your own word. Maximum of 3 answers possible.

o Clear
o Professional
o Crowded
o Attractive
o Boring
o Confusing
o Reliable
o Gloomy
o Inviting
o Fill in a word of your own:..................................

14. Please indicate the extent to which you agree or disagree with the statements below. We would like to have your opinion. There are no right or wrong answers.

	**Totally disagree**				**Totally agree**
**1. The slideshow can help me discuss complementary medicine with my healthcare provider at the hospital**	-	-	-	-	-
**2. I learn more about complementary medicine through the slideshow**	-	-	-	-	-
**3. I have no need to use the slideshow**	-	-	-	-	-
**4. I find the examples of complementary medicine in the slideshow appropriate**	-	-	-	-	-
**5. The slideshow gives me a sense of control over the conversation with my healthcare provider**	-	-	-	-	-
**6. I think I would use the slideshow before discussing**	-	-	-	-	-

**complementary medicine with my healthcare provider**					
**7. The slideshow diminishes fear of discussing complementary medicine with my healthcare provider**	-	-	-	-	-
**8. The slideshow appeals to me**	-	-	-	-	-
**9. The slideshow is useful for my family and friends**	-	-	-	-	-
**10. The slideshow is useful for other patients with cancer**	-	-	-	-	-

15. The conversation aid is...
On each line, click the circle closest to your opinion.

Clear	o	o	o	o	o	Unclear
Useful	o	o	o	o	o	Not useful
Complete	o	o	o	o	o	Incomplete
Amateuristic	o	o	o	o	o	Professional
Fast	o	o	o	o	o	Slow
Educational	o	o	o	o	o	Not educational
Reliable	o	o	o	o	o	Unreliable
Difficult	o	o	o	o	o	Simple
Reassuring	o	o	o	o	o	Distressing
Emotional	o	o	o	o	o	Not emotional
